# Muscadine Grape Skin Extract Induces an Unfolded Protein Response-Mediated Autophagy in Prostate Cancer Cells: A TMT-Based Quantitative Proteomic Analysis

**DOI:** 10.1371/journal.pone.0164115

**Published:** 2016-10-18

**Authors:** Liza J. Burton, Mariela Rivera, Ohuod Hawsawi, Jin Zou, Tamaro Hudson, Guangdi Wang, Qiang Zhang, Luis Cubano, Nawal Boukli, Valerie Odero-Marah

**Affiliations:** 1 Center for Cancer Research and Therapeutic Development, Department of Biological Sciences, Clark Atlanta University, Atlanta, GA, 30314, United States of America; 2 Department of Microbiology and Immunology, School of Medicine, Universidad Central del Caribe, Bayamon, PR, 00956, United States of America; 3 Department of Medicine, Howard University, Washington, DC, 20060, United States of America; 4 Department of Chemistry, Xavier University, New Orleans, LA, 70125, United States of America; University of Hong Kong, HONG KONG

## Abstract

Muscadine grape skin extract (MSKE) is derived from muscadine grape (*Vitis rotundifolia*), a common red grape used to produce red wine. Endoplasmic reticulum (ER) stress activates the unfolded protein response (UPR) that serves as a survival mechanism to relieve ER stress and restore ER homeostasis. However, when persistent, ER stress can alter the cytoprotective functions of the UPR to promote autophagy and cell death. Although MSKE has been documented to induce apoptosis, it has not been linked to ER stress/UPR/autophagy. We hypothesized that MSKE may induce a severe ER stress response-mediated autophagy leading to apoptosis. As a model, we treated C4-2 prostate cancer cells with MSKE and performed a quantitative Tandem Mass Tag Isobaric Labeling proteomic analysis. ER stress response, autophagy and apoptosis were analyzed by western blot, acridine orange and TUNEL/Annexin V staining, respectively. Quantitative proteomics analysis indicated that ER stress response proteins, such as GRP78 were greatly elevated following treatment with MSKE. The up-regulation of pro-apoptotic markers PARP, caspase-12, cleaved caspase-3, -7, BAX and down-regulation of anti-apoptotic marker BCL2 was confirmed by Western blot analysis and apoptosis was visualized by increased TUNEL/Annexin V staining upon MSKE treatment. Moreover, increased acridine orange, and LC3B staining was detected in MSKE-treated cells, suggesting an ER stress/autophagy response. Finally, MSKE-mediated autophagy and apoptosis was antagonized by co-treatment with chloroquine, an autophagy inhibitor. Our results indicate that MSKE can elicit an UPR that can eventually lead to apoptosis in prostate cancer cells.

## Introduction

Natural products with anticancer activities have gained increased attention due to their favorable safety and efficacy profiles as possible therapeutic agents that are not toxic to the surrounding healthy tissue. Of special interest is to determine how these novel compounds affect the endoplasmic reticulum (ER), an essential organelle for proper protein folding. Different physiological and pathological conditions can perturb protein folding in the ER, leading to a condition known as ER stress. ER stress activates the unfolded protein response (UPR), a complex intracellular signal transduction pathway that reestablishes ER homeostasis through adaptive mechanisms involving the stimulation of autophagy. The goal of the UPR is to restore optimal ER function. However, when persistent, ER stress can switch from the cytoprotective functions of UPR and autophagy to promote cell death mechanisms [1].

Flavonoids from naturally rich fruits modulate cell cycles, induce apoptosis, and inhibit extracellular regulated kinase (ERK) phosphorylation; mechanism that are linked to their confirmed anti-carcinogenic, anti-proliferative, co-chemotherapeutic, and estrogenic effects [2]. Numerous reports support the fact that many natural products play a positive role in cancer prevention and treatment by adjusting the oxidative stress response, inhibiting cancer cell proliferation and modulating apoptosis and autophagy; mechanisms that control cellular fate by regulating the turnover of organelles and proteins [3,4]. In general, autophagy blocks the induction of apoptosis, while apoptosis-associated caspase activation shuts off the autophagic process. In spite of its role as a self-digestion mechanism, autophagy is mainly activated to protect against cell death [5]. However, just like in the case of the UPR, stimulation of autophagy can be required to activate the cell death machinery under certain circumstances [6].

Anthocyanin compounds, the main bioactive components found in muscadine grape skin extract (MSKE) from the muscadine grape (*Vitis rotundifolia*), inhibit prostate cancer cell growth and promote apoptosis *in vitro* without toxicity to normal prostate epithelial cells [7]. Unlike other grape varieties, the phytochemical constituents of muscadine grapes have a higher concentration of anthocyanin 3,5-diglucosides, ellagic acid, and ellagic acid precursors [8]. In the case of purple skinned muscadine grapes, the anthocyanins are primarily delphinidin-3,5-diglucoside; cyanidin-3,5-diglucoside; and petunidin-3,5-diglucoside [8]. Anthocyanin 3,5 diglucosides are known to inhibit invasion in human hepatoma cells [9], and induce apoptosis and inhibit invasion in colorectal cancer cells [10]. MSKE has been shown to decrease tumor incidence, promote growth inhibition and stimulate cell death in various human cancer cell lines [7]. It can also revert the epithelial mesenchymal transition process [11]. Furthermore, the *in vivo* therapeutic effects of MSKE against prostate adenocarcinoma (PCa) are currently being investigated in a clinical trial [12].

In this study, proteomics, Western blot, acridine orange, Annexin V and TUNEL staining were used to determine global effects of MSKE on prostate cancer cells using C4-2 cells as a model. Our results revealed that MSKE regulated the expression of proteins important for ER stress response (GRP78, PDIA4, PDIA6, EIF2, EIF4 and Ire-1 alpha) and autophagy (ACIN1, PI4KA, PGK2 and MTDH). Pro-apoptotic markers were up-regulated, while anti-apoptotic protein BCL2 was down-regulated in the presence of MSKE; these effects were antagonized by co-treatment with chloroquine, suggesting that MSKE may promote ER stress-driven autophagic response leading to apoptosis.

## Materials and Methods

### Cell Culture, Reagents and Antibodies

C4-2 human prostate cells were grown in RPMI (Lonza, Alpharetta, GA) supplemented with 10% fetal bovine serum (Atlanta Biologicals, Flowery Branch, GA) and 1 × penicillin-streptomycin solution (Mediatech, Manassas, VA) at 37°C in a humidified incubator with 5% CO_2_. MSKE, which is composed mainly of anthocyanins, was prepared as previously described [7]. The protease inhibitor cocktail was purchased from Roche Molecular Biochemicals (Indianapolis, IN) and used according to the manufacturer’s instructions. Chloroquine (autophagy inhibitor) was purchased from Sigma Aldridge.(St. Louis, MO). Annexin V/cell death apoptosis kit was purchased from Thermo Fisher Scientific (Waltham, MA).

### Gel-free Isobaric Labeling Tandem Mass Tag Quantitative Proteomic Profiling of C4-2 Cells Treated with MSKE

#### Cell lysis and protein extraction

Cells were plated on 150 cm^2^ culture plates at a cell density of 5 × 10^6^ and treated the following day with 20 μg/ml MSKE for 72 h. Cells treated with 0.1% ethanol were used as controls. Proteins were extracted with RIPA buffer (1.5 M Tris pH 8.8, 1.75 g NaCl, 2 mL sodium dodecyl sulfate 10%, 2 mL Triton X-100; all reagents from Thermo Fisher Scientific, Waltham, MA). The cells were incubated on ice for 30 min, followed by 5 min sonication and centrifugation at 20,000 rpm for 5 min in preparation for protein extraction. Protein concentration was calculated on microtiter plates by measuring the absorbance at 595 nm of samples containing a commercial protein assay (Bio-Rad Laboratories, Hercules, CA) supplemented with 10 μL of phosphatase inhibitor cocktail and 10 μL of protease inhibitor cocktail (Roche Molecular Biochemicals, Indianapolis, IN).

#### Reduction, alkylation, and trypsin digestion

Aliquots with 100 mg of proteins from each sample were added to 100 ml of 200 mM triethyl ammonium bicarbonate TEAB (Sigma-Aldrich, St. Louis, MO). Reduction was performed by adding 5 ml of 200 mM tris (2-carboxyethyl) phosphine TCEP (Sigma-Aldrich, St. Louis, MO) to each replicate and incubating for 1 h at 55°C. Alkylation was carried out by adding 5 ml of 375 mM iodoacetamide (Bio-Rad Laboratories, Hercules, CA) to each sample and incubating for 30 min at room temperature. After alkylation, 1 ml of pre-chilled acetone was added and precipitation was allowed to proceed for 3 h at 20°C. Acetone-precipitated protein pellets were suspended in 100 ml of 200 mM TEAB and digested overnight at 37°C with 2.5 μg of sequencing grade modified trypsin (Promega Corp., Madison, WI) as previously described [13,14].

### Isobaric Labeling with Tandem Mass Tag

Tandem mass tag TMT with varying molecular weights *(126 ~ 131)* (Thermo Scientifc, Waltham, MA) were applied as isobaric labels for the comparison of differential protein expression between C4-2 cells treated with ethanol (0.1%) and C4-2 treated with 20 μg/ml MSKE. Six digested samples were individually labeled with TMT^6^ reagents according to the manufacturer’s protocols. Three control (ethanol-treated) samples: TMT-126 (batch 1), TMT-127 (batch 2), and TMT-128 (batch3); and three MSKE-treated samples: TMT-129 (batch 1), TMT-130 (batch 2), and TMT-131 (batch3) were used in the studies. The labeled peptide mixtures were combined at equal ratios.

#### Fractionation of labeled peptide mixture by using a strong cation exchange column

The TMT-labeled peptide mixture was fractionated with a strong cation exchange SCX column (Thermo Fisher Scientific, Waltham, MA) on a Shimadzu 2010 high performance liquid chromatography (HPLC) equipped with an ultraviolet detector (Shimadzu, Columbia, MD) and a mobile phase consisting of buffer A (5 mM KH_2_PO_4_, 25% acetonitrile, pH 2.8) and buffer B (buffer A plus 350 mM KCl). The column was equilibrated with buffer A for 30 min before sample injection. A mobile phase gradient at a flow rate of 1.0 ml/min was set as follows: 1) 0 ~ 10 min: 0% buffer B; 2) 10 ~ 40 min: 0 ~ 25% buffer B; 3) 40 ~ 45 min: 25 ~ 100% buffer B; 4) 45 ~ 50 min: 100% buffer B; 5) 50 ~ 60 min: 100 ~ 0% buffer B; and 6) 60 ~ 90 min: 0% buffer B. Sixty fractions were initially collected, lyophilized, and combined into 10 final fractions based on SCX chromatogram peaks.

#### Desalination of fractionated samples

A C_18_ solid phase extraction SPE column (Thermo Fisher Scientific, Waltham, MA) was used to desalt all collected fractions as previously described [15]. Briefly, the 10 combined fractions were each adjusted to a final volume of 1 ml in a 0.25% trifluoroacetic acid TFA aqueous solution. The C_18_ SPE column was preconditioned with 1 ml acetonitrile and eluted in approximately 3 min before it was rinsed with 3 × 1 ml 0.25% TFA. The fractions were loaded on to the top of the SPE cartridge column slowly, and were reloaded once again to decrease lost peptide during column binding. Columns were washed with 4 × 1 ml 0.25% TFA aliquots before the peptides were eluted with 3 × 400 μl 80% acetonitrile/0.1% formic acid (aqueous). The eluted samples were lyophilized prior to the liquid chromatography mass spectrometry LC-MS/MS analysis.

#### LC-MS/MS analysis

Peptides were analyzed on an LTQ-Orbitrap XL (Thermo Fisher Scientific, Waltham, MA) instrument interfaced with an Ultimate 3000 Dionex LC system (Dionex, Sunnyvale, CA). High mass resolution was utilized for peptide identification and high-energy collision dissociation (HCD) was used for reporter ion quantification as previously described [15]. Briefly, the reverse phase LC system contained a peptide Cap-Trap cartridge (0.5 × 2 mm) (Michrom BioResources, Auburn, CA) and a pre-packed BioBasic C_18_ PicoFrit analytical column (75 μm i.d. × 15 cm length, New Objective, Woburn, MA) fitted with a FortisTip emitter tip. Samples were loaded onto the trap cartridge and washed with mobile phase A (98% H_2_O, 2% acetonitrile, and 0.1% formic acid) for concentration and desalting. Peptides were eluted over 180 min from the analytical column via the trap cartridge by using a linear gradient of 6 to 100% mobile phase B (20% H_2_O, 80% acetonitrile, and 0.1% formic acid) at a flow rate of 0.3 μl/min.

The Orbitrap mass spectrometer was operated in a data-dependent mode in which each full MS scan (60,000 resolving power) was followed by six MS/MS scans where the three most abundant molecular ions were dynamically selected and fragmented by collision-induced dissociation with a normalized collision energy of 35% and subsequently scanned by HCD-MS^2^ with a collision energy of 45% as previously described [15]. Only the 2+, 3+, and 4+ ions were selected for fragmentation by collision-induced dissociation and HCD.

### Database Search and TMT Quantification

The protein search algorithm SEQUEST was used to identify unique protein peptides using the Proteome Discoverer data processing software (version 1.2, Thermo Fisher Scientific, Waltham, MA). The ratios of TMT reporter ion abundances in MS/MS spectra generated by HCD from raw data sets were used For TMT quantification. Fold changes in proteins between control and treatment were calculated as previously described [15].

### Western Blot

20–30 μg of proteins extracted with RIPA buffer were analyzed by SDS PAGE and transferred to a nitrocellulose membrane. Membranes were blocked with 5% non-fat dry milk in 1 × TBST buffer (BioRad, Hercules, CA) and incubated with primary antibodies overnight at 4°C. The following primary antibodies were used at a 1:1000 dilution LC3B, cleaved caspase-3, cleaved caspase-7, BAX, and BCL2 (Cell Signaling Technologies Danvers, MA.), anti-caspase-12, Ire-1α, GRP78, DFF45, and PARP (Abcam, Cambridge, MA). Anti-β-actin (1:500, Sigma-Aldrich, St. Louis, MO) were used as a loading control. Horseradish peroxidase (HRP)-conjugated secondary antibodies (Sigma Aldrich, St Louis, MO) were used and protein bands were visualized by a chemiluminescence method using the SuperSignal West Femto Maximum Sensitivity Substrate kit (Thermo Fisher Scientific, Waltham, MA). Images were analyzed with the ImageJ image processing program version 1.50b (National Institutes of Health, Bethesda, MD) to access the differential expression of key ER stress and apoptotic markers.

### Analysis of Autophagy

Acridine Orange (AO) was used to analyze autophagy. Briefly, 5 × 10^3^ cells were plated into Nunc 8-well chamber slides (Bio-Tek Instruments, Winooski, VT). Cells were serum-starved for 4 h and treated with increasing concentrations (0 μg/mL, 5 μg/mL, 10 μg/mL or 20 μg/mL) of MSKE for 72 h. Fixation was performed with methanol/ethanol (1:1 volume) for 5 min, followed by washes with 1 × PBS. Cells were exposed to 5 μg/ml AO for 15 min at 37°C, washed with 1 × PBS, and counterstained with 1 μg/ml DAPI (Santa Cruz Biotechnology, Santa Cruz, CA) before they were fixed with Fluorogel mounting medium (Electron Microscopy Sciences, Hatfield, PA). Co-treatments with the autophagy inhibitor (20 μM chloroquine) for 72 h was also performed. Fluorescence microscopy was performed at 40 × oil magnification on a Zeiss fluorescence microscope equipped with the AxioVision (release 4.8) imaging software and the ApoTome.2 optical sections system (Carl Zeiss Microscopy GmbH, Jenna, Germany).

### Immunofluoresence for LC3B

5 × 10^3^ cells were plated into Nunc 8-well chamber slides (Bio-Tek Instruments, Winooski, VT). Cells were serum-starved for 4 h and treated with 0 μg/mL or 20 μg/mL of MSKE with or without 20 μM chloroquine co-treatment for 72 h. Fixation was performed with methanol/ethanol (1:1 volume) for 5 min, followed by washes with 1 × PBS. Subsequently, slides were incubated with primary antibody (LC3B) at 1:50dilution in Dako antibody diluent solution for 1 h at room temp. Slides were washed with 1× TBS-T (Dako, Camarillo, CA), then incubated with secondary antibody in the dark for 1 h at room temp. Anti-rabbit Oregon green 488 was utilized as a secondary, and slides were then counterstained with 1 μg/ml DAPI (Santa Cruz Biotechnology, Santa Cruz, CA) before they were fixed with Fluorogel mounting medium (Electron Microscopy Sciences, Hatfield, PA). Fluorescence microscopy was performed at 40 × oil magnification on a Zeiss fluorescence microscope equipped with the AxioVision (release 4.8) imaging software and the ApoTome.2 optical sections system (Carl Zeiss Microscopy GmbH, Jenna, Germany).

### TUNEL Assay

The terminal deoxynucleotidyl transferase (TdT) dUTP nick-end tabeling (TUNEL) assay was used to detect apoptosis. Briefly, 5 × 10^3^ cells were plated into 16-well Nunc chamber slides (Bio-Tek, Winooski, VT). Cells were serum starved for 4 h and treated with increasing concentrations of MSKE (0 μg/mL, 5 μg/mL, 10 μg/mL or 20 μg/mL) for 72 h. The cells were fixed with 4% paraformaldehyde and permeabilized with 0.1% sodium citrate and 0.1% Triton X-100. DNA fragmentation was determined by TUNEL according to the manufacturer’s instructions (Roche Applied Science, Penzberg, Germany) prior to counterstaining with 1 μg/ml DAPI (Santa Cruz Biotechnology, Santa Cruz, CA). Slides were mounted with Fluorogel mounting medium (Electron Microscopy Sciences, Hatfield, PA) and visualized under 40 × oil magnification on a Zeiss fluorescence microscope equipped with the AxioVision (release 4.8) imaging software and the ApoTome.2 optical sections system (Carl Zeiss Microscopy GmbH, Jenna, Germany).

### Annexin V/Cell Death Apoptosis Kit

Annexin V was also used to detect apoptosis. Briefly C4-2 Cells were serum starved for 4 h and treated with different concentrations of MSKE (0 μg/mL, 5 μg/mL) for 72 h, with or without 20 μM chloroquine. Apoptosis was assessed by Alexa Fluor 488 Annexin V and propidium iodide double staining and flow cytometry was performed using Accuri C6 Flow Cytometer (Accuri Cytometers Inc., Ann Arbor, MI.) according to the manufacterer instructions. Cells that stained positive for Annexin V- Alexa Fluor 488 and negative for PI (Alexa Fluor 488^+^/PI^-^) were considered to be undergoing early apoptosis; Alexa Fluor 488^+^/PI^+^ were considered as late apoptosis; Alexa Fluor 488^-^/PI^-^ considered non-apoptotic (viable). Experiments were performed in triplicate in two independent experiments, and a representative result displayed in the form of early/late apoptosis graphs.

### Statistical Analysis

Cytotoxicity assays and proteomics analyses were performed in triplicate, and similar results were obtained on at least three separate studies. Statistical analysis was performed using paired or unpaired t-tests, as appropriate, using the GraphPad PRISM v6.03 statistical software (GraphPad Software, La Jolla, CA). A *p* value of ≤ 0.05 was considered statistically significant and all data are presented as means ± standard error and range.

## Results

### Proteomic Profiling of C4-2 Cells Treated with MSKE with a Gel-Free Isobaric Labeling TMT Quantitative Proteomic Approach

**Fig 1:**
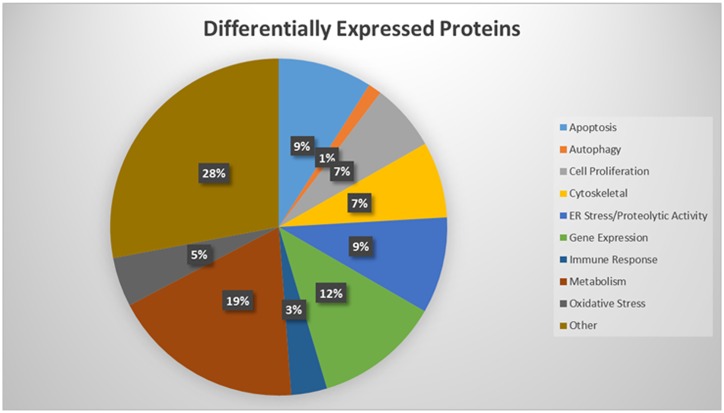
TMT labeled proteins identified by MS and organized according to biological processes. Fig 1 shows proteins up-regulated or down-regulated at least 1.2 fold in C4-2 cells treated with 20 μg/ml MSKE as identified by the MASCOT database.

The differential expression of proteins between untreated (0.1% ethanol) and treated (20 μg/ml MSKE) C4-2 cells was determined based on a gel-free isobaric labeling TMT quantitative proteomic approach for further validation and identification of novel proteins. Over 1,855 proteins were identified from control and treated C4-2 cell lysates. Among them, 465 significantly differentially expressed proteins contained TMT signals that were used to determine protein expression ratios between treated and control C4-2 cells. Proteins up-regulated or down-regulated at least 1.2 fold were organized according to biological processes (Fig 1). The detailed information including protein ID, gene name, number of amino acids, molecular weight, calculated pI, description, coverage samples, protein expression, change folds, and p-values are shown in S1 Table. Proteins that had a fold change of at least 1.2 and p-values ≤ 0.05 were closely examined and are listed in Table 1. MSKE-treated C4-2 cells expressed 254 up-regulated and 211 down-regulated proteins when compared to C4-2 control cells.

**Table 1:** Differentially expressed proteins in MSKE-treated and control cultures.

Description	Score	P-value	Gene Symbol	Σcoverage	Change Fold	MW [kDa]	calc. pI
**APOPTOSIS**							
Peptidyl-prolyl cis-trans isomerase F, mitochondrial	85.79	2.63417E-09	PPIF	21.26	1.23	22.03	9.38
Aldo-keto reductase family 1 member C1	20.02	0.009954054	AKR1C1	5.45	1.88	24.94	7.49
PAX3- and PAX7-binding protein 1	31.82	0.000657658	PAXBP1	3.13	-1.43	57.40	5.25
*annexin A4	220.76	8.39334E-23	ANXA4	20.87	-1.20	36.06	6.13
Rho guanine nucleotide exchange factor (GEF) 16	97.39	1.82266E-10	ARHGEF16	12.33	-1.29	17.10	6.13
BCL2-associated transcription factor 1	30.77	0.000837529	BCLAF1	3.64	-1.23	52.89	11.22
Cyclin-dependent kinase 5	31.84	0.000654636	CDK5	5.48	-1.20	33.28	7.66
ARL-6-interacting protein 1	647.58	1.7459E-65	CG10326	18.54	1.32	17.15	7.96
*Cytochrome c oxidase subunit 4 isoform 1, mitochondrial	57.86	1.63682E-06	COX4I1	13.02	1.28	19.56	9.51
*Cytochrome c oxidase subunit 7A2, mitochondrial	107.11	1.94405E-11	COX7A2	15.66	1.23	9.39	9.76
casein kinase 2, alpha 1 polypeptide	103.29	4.68943E-11	CSNK2A1	11.95	-1.20	45.28	7.94
*Catenin delta-1	818.43	1.43523E-82	CTNND1	19.21	-1.29	90.34	7.03
Drebrin-like protein	45.33	2.93089E-05	DBNL	3.4	-1.20	43.01	5.03
Diablo homolog, mitochondrial	29.52	0.001116863	DIABLO	37.66	1.46	8.84	5.01
Dynein light chain 2, cytoplasmic	163.12	4.87128E-17	DYNLL2	32.58	-1.23	10.34	7.37
elongation factor 1-delta isoform 4	734.76	3.34299E-74	EEF1D	36.19	1.29	28.54	4.92
Glutamate-cysteine ligase, modifier subunit, isoform CRA_a	140.93	8.07457E-15	GCLM	18.5	1.32	28.60	6.02
Growth/differentiation factor 15	188.91	1.28552E-19	GDF15	14.29	1.32	34.12	9.66
*Lactoylglutathione lyase	795.53	2.79874E-80	GLO1	55.62	-1.20	19.03	6.05
Golgi phosphoprotein 3	61.95	6.37832E-07	GOLPH3	12.85	-1.24	32.91	6.71
High mobility group protein B1	757.12	1.9406E-76	HMGB1	39.53	-1.27	24.88	5.74
lamin-B1 isoform 2	55.31	2.94217E-06	LMNB1	2.66	1.24	43.00	5.12
mitogen-activated protein kinase 3	106.76	2.10863E-11	MAPK3	9.68	-1.22	17.92	8.70
Macrophage migration inhibitory factor	316.04	2.48889E-32	MIF	55.65	-1.26	12.47	7.88
Cytochrome c oxidase subunit 2	40.84	8.24138E-05	MT-CO2	4.41	1.44	25.55	4.82
NEDD8-activating enzyme E1 regulatory subunit isoform c	86.98	2.00638E-09	NAE1	5.17	1.27	50.59	5.27
occludin	78.58	1.38788E-08	OCLN	10.5	-1.64	23.31	6.06
*Programmed cell death protein 6	428.90	1.28687E-43	PDCD6	9.63	1.31	15.57	8.19
Prohibitin	1313.07	4.9365E-132	PHB	64.34	1.23	29.79	5.76
Peptidyl-prolyl cis-trans isomerase NIMA-interacting 1	28.56	0.001393157	PIN1	9.2	-1.60	18.23	8.82
Protein kinase C delta type	88.67	1.35752E-09	PRKCD	6.51	-1.35	77.45	7.75
Phosphoserine aminotransferase	1228.55	1.397E-123	PSAT1	48.67	1.26	45.33	9.03
Tyrosine-protein phosphatase non-receptor type6	73.96	4.01791E-08	PTPN6	3.43	-1.20	66.19	7.23
*Ras-related protein Rab-21	116.33	2.32652E-12	RAB21	4.89	1.20	24.33	7.94
*Ras-related protein Rab-5A	157.52	1.76979E-16	RAB5A	12.44	1.27	22.16	8.82
*Ras-related protein Rab-5B	220.12	9.73258E-23	RAB5B	17.21	1.27	23.69	8.13
*Ras-related C3 botulinum toxin substrate 1	66.40	2.29241E-07	RAC1	11.98	1.22	21.44	8.50
Sigma non-opioid intracellular receptor 1	32.66	0.000542001	SIGMAR1	6.42	-1.21	21.00	5.45
Guanine nucleotide exchange factor VAV2	25.09	0.00309803	VAV2	1.91	-1.27	96.97	6.90
glycogen synthase kinase 3 beta	37.06	0.000196789	GSK3B	20.19	-1.25	11.56	8.02
**AUTOPHAGY**							
*apoptotic chromatin condensation inducer in the nucleus isoform 3	124.92	3.21851E-13	ACIN1	3.23	-1.60	147.30	6.73
*Protein LYRIC	25.49	0.00282488	MTDH	3.04	1.91	57.49	9.54
*Phosphoglycerate kinase 2	651.02	7.91169E-66	PGK2	20.14	-1.22	44.77	8.54
*Phosphatidylinositol 4-kinase alpha	27.37	0.001832314	PI4KA	0.93	1.19	231.17	6.87
Ubiquilin-4	29.37	0.001156112	UBQLN4	5.49	1.44	63.81	5.22
**CELL PROLIFERATION**							
ADP-ribosylation factor-like protein 8A	72.86	5.17257E-08	ARL8A	9.14	-1.37	21.40	7.77
BolA-like protein 2	150.26	9.41588E-16	BOLA2	23.03	-1.28	16.92	8.19
Calcium-binding protein 39	62.81	5.24105E-07	CAB39	6.16	-2.43	39.84	6.89
DEAD (Asp-Glu-Ala-Asp) box helicase 5	197.54	1.76228E-20	DDX5	18.82	-1.24	51.70	9.03
Deoxyribonucleoside 5'-monophosphate N-glycosidase	160.14	9.68456E-17	DNPH1	29.31	1.30	19.10	5.05
Eukaryotic peptide chain release factor GTP-binding subunit ERF3A	118.28	1.48531E-12	GSPT1	6.01	1.20	55.72	5.62
Nucleolar GTP-binding protein 1	33.06	0.000494311	GTPBP4	2.12	-1.23	60.01	9.39
*HEAT SHOCK FACTOR PROTEIN 1	81.23	7.53356E-09	HSF1	5.25	-1.46	31.92	5.38
Ragulator complex protein LAMTOR2	28.86	0.00130017	LAMTOR2	14.4	1.60	13.50	5.40
*minichromosome maintenance complex component 2	146.21	2.39378E-15	MCM2	6.81	-1.56	91.22	6.43
DNA replication licensing factor MCM3	317.45	1.79731E-32	MCM3	13.78	-1.25	85.59	6.15
DNA replication licensing factor MCM4	82.29	5.90347E-09	MCM4	4.86	-1.55	92.66	6.98
minichromosome maintenance complex component 5	184.10	3.8897E-19	MCM5	8.68	-1.51	77.54	8.60
DNA replication licensing factor MCM6	95.87	2.58985E-10	MCM6	36.14	-1.63	9.53	4.41
MCM7 isoform 2	65.31	2.94243E-07	MCM7	4.24	-1.28	60.60	6.25
N-myc downstream regulated 1	559.73	1.06484E-56	NDRG1	20.37	-1.37	35.27	6.34
Nucleolar and coiled-body phosphoprotein 1	262.61	5.48094E-27	NOLC1	11.44	1.24	73.56	9.47
protein phosphatase, Mg2+/Mn2+ dependent, 1G	333.88	4.09025E-34	PPM1G	7.75	-1.23	57.31	4.34
protein phosphatase 1 regulatory subunit 12A isoform b	148.47	1.42272E-15	PPP1R12A	3.5	1.61	105.58	5.38
protein phosphatase 1, regulatory subunit 8	85.59	2.76058E-09	PPP1R8	10.24	-1.49	22.27	5.92
Serine/threonine-protein phosphatase 2A 55 kDa regulatory subunit B alpha isoform	372.95	5.06492E-38	PPP2R2A	13.42	-1.24	51.66	6.20
Delta-3 of Serine/threonine-protein phosphatase 2A 56 kDa regulatory subunit delta	41.00	7.94328E-05	PPP2R5D	2.42	1.52	58.42	6.77
protein phosphatase 3, regulatory subunit B, alpha	50.17	9.61612E-06	PPP3R1	11.25	-1.35	18.20	4.92
protein kinase, AMP-activated, gamma 1 non-catalytic subunit	61.42	7.2062E-07	PRKAG1	7.69	-1.28	28.27	7.02
spindle pole body component 24 homolog (SPBC24), mRNA	40.85	8.22243E-05	SPC24	11.26	-1.24	17.30	4.93
Testin	32.01	0.000629506	TES	5.59	1.59	20.64	6.48
*E3 ubiquitin-protein ligase UBR5	120.37	9.17712E-13	UBR5	1.5	1.21	308.38	5.85
ZW10 protein	55.91	2.56448E-06	ZW10	2.98	1.36	76.76	6.32
**CYTOSKELETAL**							
Neuroblast differentiation-associated protein AHNAK	254.32	3.7017E-26	AHNAK	10.05	-1.24	628.70	6.15
actin-related protein 2/3 complex subunit 4 isoform b	27.12	0.001940886	ARPC4	10.26	-1.21	9.55	7.52
Actin-related protein 2/3 complex subunit 5	42.98	5.03501E-05	ARPC5	9.63	1.37	14.80	5.06
CCDC88A protein	61.95	6.37733E-07	CCDC88A	0.33	1.21	207.67	5.97
Calicin	27.87	0.001633052	CCIN	2.38	-3.15	66.54	8.18
Cytoskeleton-associated protein 5	40.21	9.52796E-05	CKAP5	4.93	1.32	22.74	8.05
CLIP-associating protein 2	105.22	3.00771E-11	CLASP2	1.81	1.31	138.78	8.34
claudin domain-containing protein 1 isoform b	28.75	0.001333521	CLDND1	5.07	1.43	31.03	6.11
Protein cordon-bleu	22.69	0.005382698	COBL	0.55	1.61	136.60	8.07
*cortactin	28.61	0.001377209	CTTN	9.81	1.69	29.60	4.91
DmX-like protein 2	24.41	0.003622	DMXL2	0.2	-1.36	339.43	6.38
DnaJ homolog subfamily B member 6	35.27	0.000297167	DNAJB6	19.7	1.25	7.88	7.40
Niban-like protein 1	147.68	1.7054E-15	FAM129B	5.73	-1.22	82.63	6.15
Filamin-B	2200.56	8.788E-221	FLNB	20.64	-1.22	275.50	5.78
Huntingtin-interacting protein K	59.27	1.18304E-06	HYPK	11.63	-1.57	14.66	4.93
Intraflagellar transport protein 27 homolog	36.74	0.000211836	IFT27	6.2	2.31	14.02	4.64
Junction plakoglobin	400.46	8.9848E-41	JUP	14.63	-1.24	81.69	6.14
Kinesin-like protein	26.05	0.002483133	KIF3C	0.89	1.91	75.75	7.15
Kinesin light chain 2	23.07	0.004931738	KLC2	1.15	1.25	57.14	8.21
Keratin, type II cytoskeletal 8	3479.34	0	KRT8	62.32	-1.27	53.67	5.59
LIM domain and actin-binding protein 1	44.21	3.79315E-05	LIMA1	3.09	1.48	43.16	8.51
macrophage erythroblast attacher	49.94	1.01391E-05	MAEA	19.63	-1.28	11.83	8.00
Microtubule-associated proteins 1A/1B light chain 3 beta 2	32.17	0.000606736	MAP1LC3B2	11.2	1.68	14.62	8.68
palmitoylated 5 (MAGUK p55 subfamily member 5)	72.66	5.42001E-08	MPP5	8.05	-1.26	17.18	6.70
Protein PRRC1	161.42	7.21439E-17	PRRC1	6.97	1.22	46.67	5.83
Radixin	176.11	2.44856E-18	RDX	7.89	1.30	68.52	6.37
Ras-related protein R-Ras	117.96	1.59848E-12	RRAS	12.84	-1.25	23.47	6.93
Na+/H+ exchange regulatory co-factor (NHERF) mRNA	98.76	1.32928E-10	SLC9A3R1	14.65	-1.42	29.42	6.80
spectrin, beta, non-erythrocytic 2	35.29	0.000295801	SPTBN2	1.61	-1.48	105.60	7.75
tubulin, beta 6 class V	1231.71	6.7485E-124	TUBB6	55.67	-1.22	44.57	4.93
*Alpha-taxilin	32.11	0.000615177	TXLNA	2.56	1.37	61.85	6.52
**ER STRESS**							
Cystathionine gamma-lyase	306.48	2.24785E-31	CTH	13.3	1.47	39.48	6.90
*Translation initiation factor eIF-2B subunit alpha	33.77	0.000419759	EIF2B1	4.61	1.51	16.92	6.90
Protein Niban	67.07	1.96515E-07	FAM129A	3.02	1.44	103.07	4.78
Mesencephalic astrocyte-derived neurotrophic factor	139.84	1.03761E-14	MANF	25.27	1.29	20.69	8.69
B-cell receptor-associated protein 31	389.58	1.10175E-39	BCAP31	22.36	1.21	27.97	8.44
*Eukaryotic translation initiation factor 4E-binding protein 1	124.81	3.30025E-13	EIF4EBP1	15.25	1.28	12.57	5.48
Endoplasmic reticulum resident protein 29	364.95	3.20115E-37	ERP29	26.05	1.30	28.98	7.31
Endoplasmin	6243.82	0	HSP90B1	52.93	1.30	92.41	4.84
*78 kDa glucose-regulated protein	7582.64	0	HSPA5	60.24	1.67	72.29	5.16
*60 kDa heat shock protein, mitochondrial	19539.31	0	HSPD1	82.02	1.23	61.02	5.87
Protein disulfide-isomerase	1239.55	1.1085E-124	P4HB	49.46	1.38	32.13	8.51
Protein disulfide-isomerase	3026.26	2.3635E-303	P4HB	58.07	1.37	57.08	4.87
*Protein disulfide-isomerase A4	3139.65	0	PDIA4	53.18	1.21	72.89	5.07
*Protein disulfide-isomerase A6	1106.04	2.4891E-111	PDIA6	31.35	1.26	47.81	5.08
*Thioredoxin-related transmembrane protein 2	30.71	0.00084918	TMX2	10.47	1.22	29.62	8.65
E3 UFM1-protein ligase 1	42.96	5.05825E-05	UFL1	3.93	1.20	57.55	6.30
endoplasmic reticulum-resident ubiquitin-like domain member 1 protein (Fragment)	59.25	1.1885E-06	HERPUD1	8.19	1.37	26.20	5.77
**GENE EXPRESSION**							
acidic leucine-rich nuclear phosphoprotein 32 family member E isoform 3	313.17	4.81956E-32	ANP32E	22.27	-1.43	25.11	3.74
Ataxin-2-like protein	434.72	3.37392E-44	ATXN2L	1.14	1.54	102.83	8.63
Barrier-to-autointegration factor	382.78	5.27164E-39	BANF1	40.45	-1.36	10.05	6.09
C5orf38 protein	34.05	0.000393587	C5orf38	8.15	1.33	14.68	11.27
chromobox homolog 1	157.35	1.8397E-16	CBX1	24.4	-1.22	19.31	5.02
CD81 molecule	122.31	5.88048E-13	CD81	21.51	-1.24	10.09	4.59
Coiled-coil-helix-coiled-coil-helix domain-containing protein 2, mitochondrial	45.54	2.79254E-05	CHCHD2	11.26	1.52	15.50	9.22
UMP-CMP kinase isoform a	632.76	5.29998E-64	CMPK1	25	-1.22	25.84	7.97
Cleavage and polyadenylation specificity factor subunit 7	48.60	1.37945E-05	CPSF7	10.55	1.32	26.13	9.51
Probable ATP-dependent RNA helicase DDX6	89.47	1.13097E-09	DDX6	8.28	1.24	54.38	8.66
Enhancer of mRNA-decapping protein 4	349.20	1.20135E-35	EDC4	11.86	-1.25	109.66	5.99
Elongation factor 1-beta	352.91	5.11228E-36	EEF1B2	44.89	1.20	24.75	4.67
Zinc phosphodiesterase ELAC protein 2	37.70	0.000169824	ELAC2	3.79	-1.20	56.06	7.43
EWS RNA-binding protein 1	149.95	1.01187E-15	EWSR1	9.42	-1.36	32.16	9.82
G-rich sequence factor 1 isoform 2	68.69	1.35317E-07	GRSF1	6.92	-1.34	36.59	5.69
general transcription factor IIA, 1, 19/37kDa	76.72	2.12814E-08	GTF2A1	18.64	-1.60	6.74	4.72
General transcription factor IIF subunit 2	59.04	1.24847E-06	GTF2F2	5.22	-1.28	28.36	9.23
histone H2A.V isoform 2	969.32	1.16911E-97	H2AFV	16.67	1.44	12.14	10.46
Heterogeneous nuclear ribonucleoprotein A/B	289.20	1.20359E-29	HNRNPAB	17.86	1.25	30.28	7.91
heterogeneous nuclear ribonucleoprotein D (AU-rich element RNA binding protein 1, 37kDa)	555.96	2.53589E-56	HNRNPD	50.89	-2.18	12.63	8.57
insulin-like growth factor 2 mRNA binding protein 3	28.17	0.001524053	IGF2BP3	12.35	-1.20	8.89	5.78
La ribonucleoprotein domain family, member 7	58.03	1.57398E-06	LARP7	7.19	-1.43	38.18	9.32
39S ribosomal protein L12, mitochondrial	188.09	1.5512E-19	MRPL12	29.29	1.47	21.33	8.87
myb-related protein A isoform 2	28.34	0.001465548	MYBL1	1.45	1.30	78.90	6.68
Myosin Ic	575.17	3.03883E-58	MYO1C	15.53	-1.25	92.27	8.87
nuclear cap binding protein subunit 1, 80kDa	34.35	0.000367282	NCBP1	29.27	-1.30	9.18	6.77
NELF-D of Negative elongation factor C/D	34.11	0.00038815	NELFCD	1.72	-1.88	65.44	5.10
Cleavage and polyadenylation specificity factor subunit 5	63.27	4.70977E-07	NUDT21	27.31	-1.33	26.21	8.82
Phosphorylated adapter RNA export protein	29.12	0.001224616	PHAX	2.79	-1.25	44.38	5.40
phosphatidylinositol-binding clathrin assembly protein isoform 4	133.72	4.24178E-14	PICALM	8.53	-1.26	59.88	8.76
Pentatricopeptide repeat-containing protein 3, mitochondrial	86.22	2.3862E-09	PTCD3	7.69	1.28	78.50	6.42
Putative peptidyl-tRNA hydrolase PTRHD1	33.65	0.000431519	PTRHD1	17.86	-1.26	15.80	9.10
retinoblastoma binding protein 7	33.52	0.000444631	RBBP7	7.69	-1.33	46.91	5.07
RNA-binding protein 10 isoform 5	53.58	4.38531E-06	RBM10	3.92	1.51	110.30	6.28
Splicing factor 45	36.91	0.00020387	RBM17	4.99	1.20	44.93	5.97
ATP-dependent DNA helicase Q1	47.07	1.9619E-05	RECQL	2.47	-1.46	73.41	7.88
ribonuclease H2 subunit B isoform 2	30.81	0.000829851	RNASEH2B	8.17	-1.39	28.99	8.82
Ribonuclease P protein subunit p14	68.05	1.56675E-07	RPP14	16.13	-1.29	13.68	7.75
Ribonuclease P protein subunit p38	74.83	3.28852E-08	RPP38	6.36	-1.32	31.81	9.92
splicing factor 3A subunit 1 isoform 2	117.45	1.79925E-12	SF3A1	7.01	1.21	81.63	5.34
Nuclear receptor coactivator NC0A-62	31.32	0.000737904	SNW1	2.67	1.20	43.31	9.70
Splicing regulatory glutamine/lysine-rich protein 1	56.27	2.36048E-06	SREK1	1.97	1.21	59.35	10.39
Serine/arginine repetitive matrix protein 1	78.24	1.49833E-08	SRRM1	8.19	-1.24	102.27	11.84
Splicing factor arginine/serine-rich 11	25.87	0.002588213	SRSF11	6.25	1.34	13.13	6.28
Splicing factor, arginine/serine-rich 5	120.94	8.056E-13	SRSF5	25.81	-1.37	14.43	10.08
Transcription elongation factor A protein 1	91.04	7.86502E-10	TCEA1	17.28	1.29	33.95	8.38
EKC/KEOPS complex subunit TPRKB	78.14	1.53358E-08	TPRKB	12.68	-1.31	16.08	6.30
tRNA (guanine-N(1)-)-methyltransferase	46.82	2.0797E-05	TRMT5	2.95	1.33	58.21	8.62
Exportin-5	28.18	0.001521507	XPO5	12.21	-1.20	136.22	5.80
zinc finger, CCHC domain containing 8	72.69	5.3827E-08	ZCCHC8	12.28	-1.52	18.10	4.65
zinc finger protein 2 (Fragment)	27.62	0.001729816	ZNF2	0.83	1.43	118.55	6.43
Zinc finger protein 511	59.64	1.08643E-06	ZNF511	5.56	-1.51	28.25	7.66
*Zinc finger SWIM domain-containing protein 5	15.31	0.029444216	ZSWIM5	0.59	-2.63	130.55	7.18
dolichyl-diphosphooligosaccharide—protein glycosyltransferase subunit 2 isoform 2 precursor	35.79	0.000263633	RPN2	15.77	1.22	67.68	6.06
**IMMUNE RESPONSE**							
Beta-defensin 119	25.85	0.002601985	DEFB119	7.14	-1.92	9.81	8.57
F-box/LRR-repeat protein 8	27.08	0.001958845	FBXL8	6.2	1.53	14.20	6.99
Guanine nucleotide-binding protein-like 1	74.73	3.36284E-08	GNL1	4.2	1.80	28.69	4.69
HLA class II histocompatibility antigen, DQB1*0602 beta chain	25.92	0.002558586	HLA-DQB1	5.88	1.31	15.76	8.85
Interferon regulatory factor 7	203.30	4.67539E-21	IRF7	4.53	1.48	42.25	5.34
Dual specificity mitogen-activated protein kinase kinase 3	38.59	0.000138357	MAP2K3	5.66	1.33	36.15	6.25
Macrophage-expressed gene 1 protein	49.94	1.01391E-05	MPEG1	0.84	1.49	78.54	7.65
*G antigen family B member 1	116.72	2.13016E-12	PAGE1	19.86	-1.53	16.14	4.22
*Phosphoinositide 3-kinase regulatory subunit 4	24.48	0.003564511	PIK3R4	0.44	1.46	153.01	7.17
Pirin	158.71	1.3466E-16	PIR	7.24	1.31	32.09	6.92
Parathymosin	230.82	8.27378E-24	PTMS	22.55	1.44	11.52	4.16
SAM domain and HD domain-containing protein 1	60.21	9.52153E-07	SAMHD1	6.15	-1.49	69.40	7.20
single immunoglobulin and toll-interleukin 1 receptor (TIR) domain	81.02	7.90679E-09	SIGIRR	11.97	-1.33	15.50	4.86
Serine/threonine-protein kinase TBK1	60.83	8.26038E-07	TBK1	2.74	-1.39	83.59	6.79
Zinc finger CCCH-type antiviral protein 1	86.04	2.48886E-09	ZC3HAV1	3.58	-1.20	77.85	8.38
**METABOLISM**							
Kynurenine/alpha-aminoadipate aminotransferase, mitochondrial	112.09	6.18431E-12	AADAT	12.12	1.24	47.79	7.21
Abhydrolase domain-containing protein 10, mitochondrial	35.49	0.000282488	ABHD10	13.73	1.23	33.91	8.57
acyl-CoA thioesterase 9	49.12	1.22462E-05	ACOT9	9.56	-1.31	15.45	8.59
Adenylate kinase isoenzyme 1	250.33	9.27582E-26	AK1	27.84	-1.20	21.62	8.63
GTP:AMP phosphotransferase, mitochondrial isoform c	91.18	7.61682E-10	AK3	22.93	1.29	18.18	8.78
Argininosuccinate synthase	548.15	1.52978E-55	ASS1	26.22	-1.35	50.79	8.40
ATP synthase subunit beta, mitochondrial	6847.36	0	ATP5B	72.78	1.20	56.52	5.40
ATP synthase gamma chain	89.04	1.24654E-09	ATP5C1	8.8	1.32	27.50	7.42
ATP synthase subunit epsilon-like protein, mitochondrial	32.85	0.0005188	ATP5EP2	13.73	1.31	5.80	10.14
ATP synthase subunit b, mitochondrial	198.92	1.28165E-20	ATP5F1	11.72	1.36	28.89	9.36
ATP synthase, H+ transporting, mitochondrial F0 complex, subunit C1 (Subunit 9)	247.94	1.60823E-25	ATP5G1	7.07	2.35	10.19	10.40
ATP synthase-coupling factor 6, mitochondrial	78.67	1.35831E-08	ATP5J	17.59	1.24	12.58	9.52
ATP synthase subunit f, mitochondrial isoform 2d	42.39	5.76766E-05	ATP5J2	59.18	1.59	5.74	9.70
ATP synthase subunit g, mitochondrial	88.08	1.55639E-09	ATP5L	28.95	1.20	8.45	10.29
ATP synthase subunit O, mitochondrial	607.69	1.70258E-61	ATP5O	38.97	1.22	23.26	9.96
ATP synthase mitochondrial F1 complexassembly factor 2, mitochondrial	42.39	5.76766E-05	ATPAF2	21.19	-1.24	13.05	9.51
ADP-ribosyl cyclase 2	49.30	1.17453E-05	BST1	1.89	1.21	35.70	7.80
citrate lyase beta like (CLYBL), transcript variant 2, mRNA	25.67	0.002710192	CLYBL	1.99	1.43	32.83	8.29
Coatomer subunit gamma-2	296.91	2.03587E-30	COPG2	7.23	-1.21	97.56	5.81
Cubilin	26.56	0.002208005	CUBN	0.28	1.26	398.48	5.35
Lanosterol 14-alpha demethylase	31.32	0.000737904	CYP51A1	6.31	1.20	25.63	9.22
Elongation factor 1-alpha 1	2876.54	2.2169E-288	EEF1A1	17.32	1.42	50.12	8.95
Elongation factor 1-gamma	847.83	1.6494E-85	EEF1G	39.13	1.24	50.09	6.67
Eukaryotic translation initiation factor 3 subunit H	219.98	1.0051E-22	EIF3H	12.78	1.29	39.91	6.54
Eukaryotic translation initiation factor 3 subunit K	146.26	2.36701E-15	EIF3K	20.18	1.30	25.04	4.93
eukaryotic translation initiation factor 4A2	236.48	2.24941E-24	EIF4A2	40.61	-1.70	41.26	5.64
Eukaryotic translation initiation factor 5	355.38	2.89998E-36	EIF5	20.88	1.27	49.19	5.58
Mitochondrial enolase superfamily member 1	31.06	0.00078343	ENOSF1	4.29	-1.21	49.75	6.48
Squalene synthase	308.02	1.57875E-31	FDFT1	19.27	1.28	47.25	6.54
Fibronectin	84.30	3.71838E-09	FN1	1.38	-1.75	222.84	5.68
fibronectin 1 (FN1), transcript variant 5, mRNA	84.30	3.71838E-09	FN1	2.96	-1.32	111.23	6.21
Galactokinase	65.27	2.97119E-07	GALK1	10.71	1.61	42.25	6.46
N-acetylgalactosamine kinase	33.04	0.000496592	GALK2	4.15	1.39	47.60	6.19
FAD-linked sulfhydryl oxidase ALR	73.98	3.99945E-08	GFER	12.2	1.27	23.43	7.62
Glutathione S-transferase	109.04	1.24616E-11	GSTA3	12.21	-1.22	19.73	9.16
Glutathione S-transferase Mu 3	1530.92	8.0923E-154	GSTM3	56.44	-1.24	26.54	5.54
Hydroxyacylglutathione hydrolase, mitochondrial	32.51	0.000561048	HAGH	8.08	-1.26	28.84	7.33
Histone acetyltransferase type B catalytic subunit	134.87	3.25617E-14	HAT1	5.39	-1.40	39.76	5.92
Isopentenyl-diphosphate Delta-isomerase 1	75.33	2.93089E-08	IDI1	15.86	1.24	26.30	6.34
inositol-tetrakisphosphate 1-kinase	28.46	0.001425608	ITPK1	12.5	-1.58	19.45	6.39
L-lactate dehydrogenase A-like 6A	362.00	6.31588E-37	LDHAL6A	7.23	-1.28	36.48	6.99
Phospholysine phosphohistidine inorganic pyrophosphate phosphatase	113.45	4.51551E-12	LHPP	7.78	-1.23	29.15	6.15
lanosterol synthase isoform 3	54.26	3.7465E-06	LSS	3.07	1.49	74.17	6.46
Leukotriene A-4 hydrolase	45.87	2.58821E-05	LTA4H	2.36	1.46	57.26	6.06
LYR motif-containing protein 7	56.05	2.48146E-06	LYRM7	15.38	1.68	11.95	9.66
NADP-dependent malic enzyme	1228.39	1.4492E-123	ME1	20.98	1.40	64.11	6.13
NAD-dependent malic enzyme, mitochondrial isoform 2 precursor	53.87	4.10204E-06	ME2	5.01	1.52	53.55	8.54
Cob(I)yrinic acid a,c-diamide adenosyltransferase, mitochondrial	155.89	2.57529E-16	MMAB	28.4	1.31	27.37	8.60
Mannose-6-phosphate isomerase	289.43	1.1405E-29	MPI	18.68	-1.28	46.63	5.95
39S ribosomal protein L53, mitochondrial	45.67	2.70836E-05	MRPL53	13.39	1.65	12.10	8.76
Arylamine N-acetyltransferase 1	68.56	1.39222E-07	NAT1	5.86	-1.28	33.88	6.54
NADH-ubiquinone oxidoreductase 75 kDa subunit, mitochondrial	35.89	0.000257632	NDUFS1	3.27	1.61	66.88	5.38
NADH dehydrogenase [ubiquinone] iron-sulfur protein 3, mitochondrial	34.69	0.000339625	NDUFS3	19.23	1.77	8.60	8.03
nitric oxide synthase interacting protein (NOSIP), mRNA	26.13	0.002437811	NOSIP	6.63	-1.81	17.93	8.00
nucleoporin 133kDa	169.20	1.2026E-17	NUP133	7.19	-1.26	120.30	5.15
Nuclear pore complex protein Nup205	41.85	6.53131E-05	NUP205	2.83	-1.40	227.78	6.19
Nuclear pore complex protein Nup93	25.65	0.002722701	NUP93	1.15	-1.25	79.83	6.18
nucleoporin 98kDa	447.74	1.68228E-45	NUP98	6.9	-1.53	26.86	7.39
propionyl-CoA carboxylase alpha chain, mitochondrial isoform c precursor	24.30	0.003715352	PCCA	1.03	1.46	74.95	7.36
phosphoacetylglucosamine mutase isoform 3	113.80	4.17131E-12	PGM3	8.03	1.33	51.05	5.88
Arfaptin-2	31.82	0.000657658	POR	16.99	1.20	47.37	7.81
Lipid phosphate phosphohydrolase 1	29.39	0.0011508	PPAP2A	7.04	1.22	32.14	7.97
Palmitoyl-protein thioesterase 1	126.47	2.25388E-13	PPT1	9.48	-1.34	34.17	6.52
glucosidase 2 subunit beta isoform 2	450.79	8.33915E-46	PRKCSH	20.19	1.28	59.14	4.42
Prosaposin	1508.14	1.5356E-151	PSAP	21.76	-1.33	58.07	5.17
Ran GTPase activating protein 1	115.42	2.87351E-12	RANGAP1	9.02	-1.35	57.74	4.53
rap1 GTPase-GDP dissociation stimulator 1 isoform 6	90.06	9.85348E-10	RAP1GDS1	11.82	-1.20	56.50	5.44
60S acidic ribosomal protein P2	1310.74	8.4427E-132	RPLP2	70.43	-1.29	11.66	4.54
SUMO-activating enzyme subunit 1	481.73	6.71984E-49	SAE1	18.79	-1.25	38.43	5.30
Sec1 family domain-containing protein 1	72.62	5.47514E-08	SCFD1	6.13	1.24	51.51	6.96
Succinate dehydrogenase [ubiquinone] flavoprotein subunit, mitochondrial	344.95	3.19778E-35	SDHA	9.61	1.26	63.53	7.24
Protein transport protein Sec24B	38.99	0.000126098	SEC24B	0.89	-1.48	133.54	7.39
serine hydroxymethyltransferase, mitochondrial isoform 3	1864.72	3.3762E-187	SHMT2	49.28	1.24	53.42	8.15
Monocarboxylate transporter 1	106.48	2.24967E-11	SLC16A1	10.42	-1.29	51.80	8.50
Tricarboxylate transport protein, mitochondrial	73.34	4.63657E-08	SLC25A1	7.4	1.20	33.99	9.89
Calcium-binding mitochondrial carrier protein Aralar2	64.84	3.27874E-07	SLC25A13	8.11	1.21	62.21	9.01
solute carrier organic anion transporter family member 1C1 isoform 3	30.95	0.000803526	SLCO1C1	0.9	1.52	73.22	8.85
Translocon-associated protein subunit alpha	271.19	7.59987E-28	SSR1	8.88	1.25	29.36	4.61
Translocon-associated protein subunit delta	141.51	7.06092E-15	SSR4	17.5	1.40	13.06	7.50
UDP-N-acetylhexosamine pyrophosphorylase	80.69	8.52774E-09	UAP1	2.97	1.23	56.99	6.38
UTP—glucose-1-phosphate uridylyltransferase	25.07	0.003111716	UGP2	4.02	1.23	55.64	7.88
Cytochrome b-c1 complex subunit 9	57.97	1.5948E-06	UQCR10	38.1	1.31	7.30	9.47
Cytochrome b-c1 complex subunit 7	94.76	3.34256E-10	UQCRB	21.62	1.22	13.52	8.78
**OXIDATIVE STRESS**							
Cytoplasmic aconitate hydratase	179.53	1.11364E-18	ACO1	6.97	-1.36	98.34	6.68
*Retinal dehydrogenase 1	385.17	3.03767E-39	ALDH1A1	17.95	1.32	42.57	5.80
*Fatty aldehyde dehydrogenase	262.18	6.06001E-27	ALDH3A2	12.99	1.23	54.81	7.88
Alkylated repair protein alkB	78.29	1.48152E-08	ALKBH5	5.48	-1.41	43.02	9.16
aspartyl/asparaginyl beta-hydroxylase isoform f	131.03	7.88327E-14	ASPH	4.39	2.23	83.22	4.92
Cytochrome b561	86.52	2.22693E-09	CYB561	4.38	1.45	27.54	8.56
Cytochrome b5	126.09	2.45962E-13	CYB5A	42.86	-1.22	11.26	5.14
*Dehydrogenase/reductase SDR family member 1	67.71	1.69434E-07	DHRS1	6.07	1.31	33.89	7.83
Glycine N-methyltransferase	39.20	0.000120226	GNMT	2.71	-1.25	32.72	7.02
MOSC domain-containing protein 1, mitochondrial	40.11	9.7499E-05	MARC1	13.89	-1.23	28.42	7.31
3-mercaptopyruvate sulfurtransferase	72.72	5.34203E-08	MPST	14.48	1.23	33.16	6.60
Bifunctional methylenetetrahydrofolate dehydrogenase/cyclohydrolase, mitochondrial	33.26	0.000472063	MTHFD2	5.41	2.00	23.85	9.76
Myb-binding protein 1A	64.54	3.5156E-07	MYBBP1A	0.83	-1.29	148.76	9.28
NADH dehydrogenase [ubiquinone] iron-sulfur protein 8, mitochondrial	40.43	9.05733E-05	NDUFS8	8.18	1.86	12.40	9.98
*NAD(P)H dehydrogenase, quinone 1 (NQO1), transcript variant 3, mRNA	513.20	4.78822E-52	NQO1	52.48	1.28	22.78	8.50
Prolyl 4-hydroxylase subunit alpha-2	36.08	0.000246604	P4HA2	2.25	1.41	60.59	5.71
Protoporphyrinogen oxidase	87.40	1.8197E-09	PPOX	6.98	1.26	46.81	7.31
Pyrroline-5-carboxylate reductase 2	93.93	4.04761E-10	PYCR2	9.38	1.23	33.62	7.77
Retinol dehydrogenase 14	64.68	3.40408E-07	RDH14	7.14	1.44	36.84	8.79
*Sideroflexin-1	318.98	1.26412E-32	SFXN1	32.61	1.21	35.60	9.07
*STEAP family member 2, metalloreductase	90.06	9.86169E-10	STEAP2	6.7	-1.47	45.95	8.91
**PROTEOLYTIC**							
ADAM metallopeptidase domain 9	26.77	0.002103778	ADAM9	3.82	-1.34	69.28	6.65
asparagine synthetase [glutamine-hydrolyzing] isoform b	2335.19	3.0244E-234	ASNS	38.89	1.31	62.13	7.06
Cyclin-dependent kinase 1	29.04	0.001247384	CDK1	8.33	-1.57	27.49	7.06
*Collagen type IV alpha-3-binding protein	48.53	1.40281E-05	COL4A3BP	1.67	-1.40	67.96	5.47
Cystatin-B	190.50	8.90894E-20	CSTB	24.49	-1.82	11.13	7.56
Farnesyl pyrophosphate synthetase like-4 protein (Fragment)	70.77	8.36775E-08	FDPS	8.62	-3.10	39.63	4.98
PSMA' of Glutamate carboxypeptidase 2	414.93	3.21326E-42	FOLH1	9.24	-1.46	77.95	6.67
Glucosamine—fructose-6-phosphate aminotransferase [isomerizing] 2	148.17	1.52319E-15	GFPT2	6.6	-1.87	76.88	7.37
Minor histocompatibility antigen H13	60.58	8.74071E-07	HM13	6.57	1.21	36.79	6.68
*Hypoxia up-regulated protein 1	2096.90	2.0428E-210	HYOU1	28.53	1.21	111.27	5.22
Leucyl-cystinyl aminopeptidase	91.92	6.43211E-10	LNPEP	2.78	-1.26	114.99	5.78
Mucosa-associated lymphoid tissue lymphoma translocation protein 1	29.41	0.001145513	MALT1	1.97	-1.47	91.02	5.91
E3 ubiquitin-protein ligase MARCH5	27.45	0.001798871	MARCH5	3.6	1.69	31.21	8.70
Neprilysin	670.63	8.64906E-68	MME	20	-1.33	85.46	5.73
neural precursor cell expressed, developmentally down-regulated 8	101.64	6.8544E-11	NEDD8	40	-1.34	5.86	6.70
aminopeptidase NPEPL1 isoform 3	158.36	1.45823E-16	NPEPL1	4.84	-1.22	50.50	6.52
Ubiquitin thioesterase OTUB2	229.61	1.09388E-23	OTUB2	7.02	2.11	12.93	9.29
*OTU domain-containing protein 6B	102.58	5.52077E-11	OTUD6B	14.58	-1.37	21.79	5.10
Proteasome subunit beta type-6	117.00	1.9941E-12	PSMB6	25.1	1.20	25.34	4.92
E3 ubiquitin/ISG15 ligase TRIM25	166.22	2.38646E-17	TRIM25	7.14	-1.22	70.93	8.09
Transcription intermediary factor 1-beta	62.59	5.50752E-07	TRIM28	33.05	-1.23	88.49	5.77
*Ubiquitin-conjugating enzyme E2 variant 2	369.35	1.16185E-37	UBE2V2	60.61	1.56	11.27	5.52
E3 ubiquitin-protein ligase UBR2	39.23	0.000119399	UBR2	4	-1.27	66.01	7.42
Deubiquitinating protein VCIP135	30.73	0.000845279	VCPIP1	0.57	-1.73	134.24	7.20
X-prolyl aminopeptidase (Aminopeptidase P) 1, soluble	38.35	0.000146218	XPNPEP1	3.26	1.41	62.10	5.73
**OTHER**							
Armadillo repeat containing 2	36.31	0.000233884	ARMC2	9.41	2.12	9.41	6.92
UPF0556 protein C19orf10	33.48	0.000448745	C19orf10	13.87	1.27	18.78	6.68
CDGSH iron-sulfur domain-containing protein 3, mitochondrial	34.93	0.000321366	CISD3	10.24	1.24	14.21	10.55
Protein FAM136A	28.08	0.001555966	FAM136A	6.52	1.30	15.63	7.61
hematological and neurological expressed 1	60.68	8.55067E-07	HN1	9.35	-1.34	11.62	8.47
kelch-like protein 35	27.78	0.001667247	KLHL35	1.2	-1.35	62.85	7.83
Leucine-rich repeat-containing protein 47	55.50	2.81838E-06	LRRC47	2.57	1.43	63.43	8.28
Nucleoside diphosphate-linked moiety X motif 22	1000.35	9.2264E-101	NUDT22	2.12	1.51	30.57	11.56
Regulator of G-protein signaling 10	36.15	0.000242661	RGS10	10.18	-1.31	19.60	5.87
Selenoprotein M	28.07	0.001559553	SELM	9.66	-1.34	16.17	5.54
Serine/threonine-protein phosphatase 4 regulatory subunit 3A	76.44	2.27171E-08	SMEK1	3.15	-1.21	61.98	4.81
Protein kish-A	37.02	0.000198609	TMEM167A	12.5	1.28	8.05	8.95
Transmembrane protein 205	34.49	0.000355631	TMEM205	8.99	-1.37	21.18	8.62
Transmembrane protein 238	42.68	5.39511E-05	TMEM238	10.23	1.40	18.03	11.53
Transmembrane protein 87A	55.86	2.59243E-06	TMEM87A	9.11	-1.33	56.74	5.69
182 kDa tankyrase-1-binding protein	106.18	2.40763E-11	TNKS1BP1	2.6	-1.46	181.69	4.86
UDP-N-acetylhexosamine pyrophosphorylase-like protein 1	31.60	0.000691831	UAP1L1	1.38	-1.24	56.99	6.32
zinc finger CCCH-type containing 18	65.78	2.64005E-07	ZC3H18	5.42	-1.87	39.04	4.89
Leucine-rich repeat-containing protein 59	503.47	4.49936E-51	LRRC59	20.52	1.22	34.91	9.57
rRNA 2'-O-methyltransferase fibrillarin	180.12	9.71673E-19	FBL	11.53	-1.33	33.76	10.18
Clustered mitochondria protein homolog	54.60	3.46545E-06	CLUH	2.14	1.20	146.58	6.13
ATP-dependent RNA helicase DDX31	282.98	5.03956E-29	DDX31	0.97	1.24	80.89	9.83
Neurobeachin-like protein 2	23.89	0.004083194	NBEAL2	0.43	1.66	282.68	6.54
NHP2 ribonucleoprotein	351.04	7.86182E-36	NHP2	33.33	-1.22	15.01	9.25
WD repeat protein 55	59.96	1.00925E-06	WDR55	21.74	-1.43	12.54	6.74
Nucleobindin-2	526.89	2.04772E-53	NUCB2	27.86	1.24	50.16	5.12
1-phosphatidylinositol-4,5-bisphosphate phosphodiesterase eta-1	49.48	1.12644E-05	PLCH1	0.8	1.38	114.43	7.64
Ankyrin repeat domain-containing protein 5	84.77	3.3358E-09	ANKEF1	1.42	1.33	86.61	8.28
Calmodulin	383.67	4.29621E-39	CALM	38.26	-1.40	16.83	4.22
Calcyclin-binding protein	64.99	3.17122E-07	CACYBP	13.16	1.30	26.19	8.25
calcium/calmodulin-dependent protein kinase kinase 2, beta	41.30	7.42096E-05	CAMKK2	6.41	-1.48	38.59	6.30
Calmodulin-regulated spectrin-associated protein 3	25.23	0.002999163	CAMSAP3	0.48	1.56	134.67	8.35
Calnexin	1219.69	1.0747E-122	CANX	23.14	1.21	67.53	4.60
Calcium-regulated heat stable protein 1	181.64	6.85365E-19	CARHSP1	10.88	-1.46	15.88	8.21
S-adenosylmethionine synthase isoform type-2	22.48	0.00564937	MAT2A	3.98	1.62	19.46	9.10
40S ribosomal protein S28	416.48	2.24773E-42	RPS28	30.43	1.65	7.84	10.70
*Tetraspanin	48.28	1.48594E-05	TSPAN13	6.29	-2.11	17.75	6.29
Desmoglein-2	112.81	5.23745E-12	DSG2	7.6	1.23	122.22	5.24
Integrin alpha-10	26.55	0.002213095	ITGA10	0.78	1.26	112.47	6.68
Liprin-alpha-1	36.20	0.000239776	PPFIA1	1.6	-1.26	133.89	6.25
WD repeat-containing protein 61	46.62	2.17624E-05	WDR61	6.56	-1.20	33.56	5.47
Syntaxin-12	84.27	3.74231E-09	STX12	6.88	1.63	31.62	5.59
*Dihydropyrimidinase-related protein 5	31.72	0.00067262	DPYSL5	3.68	1.67	20.85	6.42
PDZ domain-containing protein GIPC1 isoform 2	33.42	0.000454988	GIPC1	7.2	-1.30	26.06	5.57
Crk-II of Adapter molecule crk	168.48	1.41897E-17	CRK	7.57	1.26	33.81	5.55
Transducin-like enhancer protein 3	30.90	0.000812831	TLE3	2.11	-1.45	82.17	7.20
Platelet-activating factor acetylhydrolase IB subunit gamma	113.69	4.27563E-12	PAFAH1B3	21.65	1.34	25.72	6.84
Histone H3.3	828.78	1.32475E-83	H3F3A	44.85	1.29	15.32	11.27
Histone H1.5	536.77	2.10286E-54	HIST1H1B	21.24	2.49	22.57	10.92
Histone H1.2	1007.80	1.6588E-101	HIST1H1C	30.99	2.29	21.35	10.93
Histone H1.4	1069.83	1.0408E-107	HIST1H1E	30.59	2.89	21.85	11.03
Histone H2B type 1-C/E/F/G/I	1342.18	6.0583E-135	HIST1H2BC	62.7	1.84	13.90	10.32
Histone H3.1	1117.98	1.5924E-112	HIST1H3A	44.85	1.54	15.39	11.12
Histone H4	1317.99	1.5896E-132	HIST1H4A	52.43	1.85	11.36	11.36
Histone H2A type 2-C	969.32	1.16911E-97	HIST2H2AC	55.04	1.97	13.98	10.90
Histone H2B type 2-E	1489.58	1.1021E-149	HIST2H2BE	62.7	1.86	13.91	10.32
Histone H3.2	1363.47	4.4975E-137	HIST2H3A	44.85	1.82	15.38	11.27
Histone H2A.x	516.44	2.27136E-52	H2AFX	59.44	1.22	15.14	10.74
replication factor C subunit 5 isoform 4	34.07	0.000391742	RFC5	2.67	2.24	38.14	7.80
small ubiquitin-related modifier 2 isoform b precursor	154.56	3.50042E-16	SUMO2	16.9	-1.39	8.11	5.41
deoxyuridine 5'-triphosphate nucleotidohydrolase, mitochondrial isoform 3	160.14	9.68456E-17	DUT	21.99	1.20	15.39	6.57
Single-stranded DNA-binding protein, mitochondrial	882.96	5.06197E-89	SSBP1	53.38	1.22	17.25	9.60
WD repeat-containing protein 72	21.02	0.007906786	WDR72	0.54	1.73	123.35	6.67
Alpha-2-HS-glycoprotein	51.23	7.53356E-06	AHSG	4.9	2.19	27.34	6.52
Stonin-2	37.61	0.00017338	STON2	0.99	1.21	101.10	5.39
protein arginine methyltransferase 1	278.12	1.54264E-28	PRMT1	34.46	-1.37	37.68	6.15
Proline-, glutamic acid- and leucine-rich protein 1	29.98	0.001004616	PELP1	2.02	2.20	110.02	4.30
TBC1 domain family member 10B	32.11	0.000615177	TBC1D10B	0.87	-2.10	87.14	9.19
Nuclear ubiquitous casein and cyclin-dependent kinases substrate	204.85	3.27022E-21	NUCKS1	11.52	-1.23	27.28	5.08
SLD5 homolog (SLD5), mRNA	72.76	5.29663E-08	GINS4	12.84	2.13	12.05	5.74
Microsomal triglyceride transfer protein large subunit	39.58	0.000110154	MTTP	1.23	1.98	99.29	8.41
Protein FAM87A	23.60	0.004365158	FAM87A	4.9	-1.43	31.72	10.01
39S ribosomal protein L2, mitochondrial	47.97	1.5948E-05	MRPL2	2.62	1.22	33.28	11.30
39S ribosomal protein L21, mitochondrial isoform a	51.24	7.51623E-06	MRPL21	15.83	-1.20	13.74	10.07
Serpin B6	108.58	1.38613E-11	SERPINB6	11.49	-1.21	46.34	5.76
Vomeronasal type-1 receptor 5	23.95	0.00402717	VN1R5	1.96	-1.59	40.75	9.20
cilia and flagella associated protein 58	44.44	3.59536E-05	CFAP58	6.52	1.99	11.03	9.23
Membrane-associated progesterone receptor component 1	156.79	2.09251E-16	PGRMC1	22.05	1.29	21.66	4.70
FK506 binding protein12	205.15	3.05816E-21	FKBP1A	35.14	-1.37	3.96	5.78
Peptidyl-prolyl cis-trans isomerase B	548.34	1.4649E-55	PPIB	46.3	1.24	23.73	9.41
selenide, water dikinase 1 isoform 3	221.26	7.4799E-23	SEPHS1	16.2	1.24	35.46	5.21
Probable Xaa-Pro aminopeptidase 3	91.64	6.86257E-10	XPNPEP3	10.98	1.45	48.08	5.62
mitochondrial ribosomal protein L38 (MRPL38)	34.00	0.000398107	MRPL38	3.57	1.22	22.96	6.14
39S ribosomal protein L4, mitochondrial	52.08	6.19534E-06	MRPL4	15.21	-1.20	29.48	10.13
MRPL43 protein (Fragment)	88.64	1.36673E-09	MRPL43	8.97	1.23	15.95	8.66
60S ribosomal protein L32	68.10	1.54882E-07	RPL32	12.78	1.23	15.61	11.44
proteasome assembly chaperone 4 isoform c	50.67	8.57038E-06	PSMG4	24.04	-1.23	11.21	6.51
N-terminal Xaa-Pro-Lys N-methyltransferase 1	149.45	1.13471E-15	NTMT1	11.61	-1.99	25.46	5.52
STE20/SPS1-related proline-alanine-rich protein kinase	800.51	8.88891E-81	STK39	13.35	-1.24	59.60	6.29
toll interacting protein	62.64	5.44503E-07	TOLLIP	17.5	-1.27	8.65	8.35
SUMO-conjugating enzyme UBC9	186.06	2.47947E-19	UBE2I	34.18	-1.27	18.00	8.66
Putative NOL1/NOP2/Sun domain family member 5B	53.31	4.66317E-06	NSUN5P1	7.14	-1.70	16.47	9.26
THO complex subunit 2	40.48	8.95365E-05	THOC2	1.06	1.23	97.21	6.51
ATP-dependent RNA helicase DHX29	65.43	2.86418E-07	DHX29	0.73	1.28	155.14	8.09
Neuron-specific calcium-binding protein hippocalcin	79.07	1.23796E-08	HPCA	6.22	1.63	22.41	4.97
Olfactory receptor 5AC2	37.52	0.000177011	OR5AC2	2.27	-1.39	35.28	8.94
A-kinase anchor protein 8	61.79	6.61769E-07	AKAP8	2.89	1.60	76.06	5.15
Myeloid-associated differentiation marker	61.52	7.04693E-07	MYADM	15.65	1.74	15.92	8.65
*NF-kappa-B-activating protein	26.49	0.002243882	NKAP	1.45	1.65	47.11	10.11
*ATP-binding cassette sub-family A member 5	30.05	0.000988553	ABCA5	0.86	-5.84	104.30	7.12
AP-1 complex subunit mu-2	47.21	1.90108E-05	AP1M2	4.02	-1.53	48.08	8.22
AP-2 complex subunit mu-1	44.57	3.49424E-05	AP2M1	8.27	-1.20	42.67	9.39
ADP-ribosylation factor 5	967.37	1.83259E-97	ARF5	56.11	1.23	20.52	6.79
ADP-ribosylation factor guanine nucleotide-exchange factor 1 (brefeldin A-inhibited)	440.40	9.12033E-45	ARFGEF1	3.27	-1.23	142.44	6.13
ARFAPTIN 1	175.92	2.55929E-18	ARFIP1	17.14	1.25	28.40	7.72
Cellular nucleic acid-binding protein	117.58	1.74464E-12	CNBP	8.82	-1.62	18.73	7.71
Metal transporter CNNM3	22.85	0.005188	CNNM3	0.91	1.22	70.59	7.12
Protein CWC15 homolog	66.10	2.45471E-07	CWC15	4.8	-1.30	26.61	5.71
Acyl-CoA-binding protein	504.05	3.93581E-51	DBI	50.57	-1.32	10.04	6.57
Golgin subfamily A member 5	27.96	0.001599558	GOLGA5	9.4	1.32	12.84	6.04
Hook homolog 1	35.65	0.00027227	HOOK1	2.33	-1.28	79.97	5.19
UPF0459 protein C19orf50	24.35	0.003672823	KXD1	5.11	-1.64	19.66	4.89
LPS-responsive vesicle trafficking, beach and anchor containing	123.34	4.63532E-13	LRBA	1.75	-1.37	286.79	5.52
28S ribosomal protein S16, mitochondrial	56.67	2.15278E-06	MRPS16	9.49	1.38	15.34	9.50
28S ribosomal protein S17, mitochondrial	80.19	9.5737E-09	MRPS17	15.38	1.30	14.49	9.85
28S ribosomal protein S18b, mitochondrial	93.79	4.17548E-10	MRPS18B	7.91	1.50	24.34	8.56
myosin, light chain 6, alkali, smooth muscle and non-muscle	497.53	1.76602E-50	MYL6	46.67	-1.25	15.01	5.00
Myosin-Id	149.00	1.25876E-15	MYO1D	2.49	-1.30	116.13	9.39
Ran-binding protein 6	46.97	2.00815E-05	RANBP6	2.44	-1.25	124.63	5.01
Ammonium transporter Rh type B	28.58	0.001386756	RHBG	11.34	1.28	10.57	8.76
Secretory carrier-associated membrane protein 2	135.77	2.64671E-14	SCAMP2	3.95	1.68	36.63	6.10
Mitochondrial glutamate carrier 1	84.11	3.8815E-09	SLC25A22	4.64	1.21	34.45	9.29
ADP/ATP translocase 2	1271.34	7.3482E-128	SLC25A5	47.65	1.24	32.83	9.69
spermatogenesis associated 7	29.05	0.001244515	SPATA7	16.67	-1.46	4.76	10.26
Sarcalumenin	23.75	0.004221453	SRL	2.32	-1.71	49.78	6.95
Serine/threonine-protein kinase WNK1	31.00	0.000794328	WNK1	0.71	2.19	206.52	7.15

Note: The asterisk (*) indicates the proteins discussed in the manuscript.

Among the proteins that were differentially expressed, ER stress markers, such as 78 kDa glucose-regulated protein (GRP78) and protein disulfide isomerase A4 and A6 (PDIA4, PDIA6), as well as oxidative stress markers sulfiredoxin (SRXN1) and ubiquinone (NDUFS1 and NDUFS3) were up-regulated. Additional ER stress-related proteins identified among the up-regulations are eukaryotic translation initiation factor 2B (eIF2B) and eukaryotic translation initiation factor 4 binding protein (eIF4EBP1) with a respective significant fold change of 1.5 and 1.3 and the protein disulfide isomerase (PDI) A4 and A6 with a differential expression of 1.21 and 1.26, respectively. Key differentially expressed proteins induced by MSKE in C4-2 cells included those associated with apoptosis (e.g., STEAP2, PDCD6, MTDH, RAB5B, cytochrome c oxidase), autophagy (e.g., ACIN1, PI4KA, PGK2, MTDH), cytoskeleton and protein transport (e.g., cortactin, taxilin, radixin, filamin B), among others (Table 1). Furthermore, C4-2 control cells had a higher percent of anti-apoptotic proteins compared to the MSKE-treated cells, suggesting that MSKE induces an ER stress/autophagy/apoptosis signature.

### MSKE Induces Expression of ER Stress Mediated Pro-Apoptotic Response Proteins

**Fig 2:**
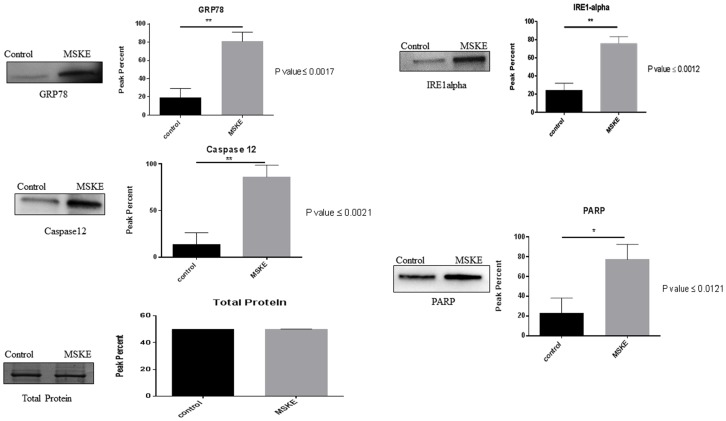
Quantitative Western blot of key ER stress markers. Western blot analysis in C4-2 cells treated with 20 μg/ml MSKE as compared to ethanol-treated controls. As a loading control total protein from Ponceau S staining was assessed. Expression of ER stress markers IRE1-alpha and GRP78 and pro-apoptotic markers DFF45, PARP and caspase-12 was analyzed and quantification of western blot analysis performed using Image J, NIH. The standard deviation was used to assess data dispersion.

Western blot was used to measure the expression of proteins associated with ER stress response. Key ER stress response markers IRE-1 alpha and GRP78 (Fig 2) were up-regulated when compared to control cells. Apoptotic markers PARP, caspase-12 and DFF45 (Fig 2) were up-regulated in cells exposed to MSKE. These data suggest that MSKE may induce ER stress and UPR-mediated apoptosis.

### MSKE Treatment Induces Autophagy

**Fig 3:**
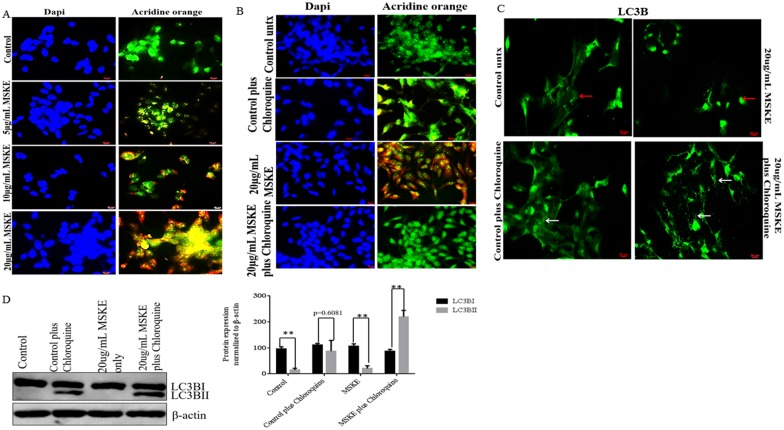
MSKE treatment induces autophagy. (A) C4-2 prostate cancer cells were treated with MSKE (0 μg/mL, 5 μg/mL, 10 μg/mL, and 20 μg/mL) for 72 h. Fixation was performed with methanol/ethanol 1:1 volume followed by washes with 1× PBS. The cells were then exposed to acridine orange (5 μg/ml) for 15 min at 37°C, followed by washes with 1× PBS, prior to counterstaining with DAPI. We observed that treatment with higher concentrations of MSKE (10 and 20μg/ml) showed extensive acridine orange leakage into the cytosol, producing a diffuse yellow color and an increase in lysosomes indicating that MSKE induces cell death via autophagy compared to control. (B) C4-2 cells were treated with MSKE, with and without 20 μM chloroquine, for 72 h. The cells were then exposed to acridine orange (5 μg/ml) for 15 min at at 37°C, followed by washes with 1× PBS, prior to counterstaining with DAPI. Chloroquine treatment reversed the effects of MSKE. (C) Immunofluorescence staining for LC3B was performed on C4-2 cells treated with MSKE plus or minus chloroquine for 72 h. (D) Western blot analysis for LC3B was performed on C4-2 cells treated with MSKE plus/minus chloroquine. Image J analysis was performed to plot the ratios of LC3BI and LC3BII relative to actin loading control. The results are representative of experiments that have been performed in triplicate at least two times independently. Graphical data represents three independent experiments; * means 0.05 > *p* value > 0.01, ** means 0.01 > *p* value > 0.001.

Since MSKE induced expression of ER stress response proteins, we sought to determine if treatment with MSKE induces autophagy. We treated C4-2 cells with 5, 10 or 20 μg/ml MSKE for 72 h and stained with acridine orange. Treatment with higher concentrations of MSKE (10 and 20 μg/mL) showed extensive acridine orange leakage into the cytosol (orange staining), indicating that MSKE induces autophagy (Fig 3A). Since the 20 μg/mL MSKE treatment showed extensive acridine orange staining we decided to see if co-treatment with chloroquine, a known autophagy inhibitor would reverse the effects of MSKE on C4-2 cells. Co-treatment with 20 μg/mL MSKE and 20 μM chloroquine lead to a decrease in acridine orange staining compared to 20 μg/mL MSKE alone, indicating that MSKE may promote autophagy (Fig 3B). To further validate a role for MSKE in autophagy, we also performed immunofluorescence and western blot with the autophagic marker, LC3B. There was increased punctate staining for LC3B in MSKE plus chloroquine co-treatments, which indicates that chloroquine prevents fusion of autophagosomes to lysosomes, and thus causes an accumulation of autophagosomes (Fig 3C). Similarly, western blot analysis showed increased LC3BII expression in MSKE plus chloroquine treatments indicating that chloroquine had inhbitied MSKE-mediated autophagy and caused accumulation of LC3BII lipidation products (Fig 3D). Of note, we did not observe increased LC3BII with MSKE treatment alone (Fig 3D), possibly due to high autophagic flux that can sometimes not be detected unless one includes an autophagy inhibitor as previously reported [16].

### MSKE Promotes Autophagy-Mediated Apoptosis

**Fig 4:**
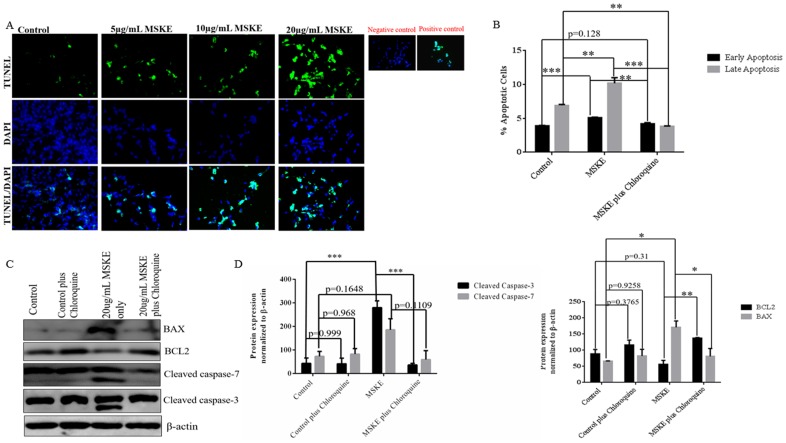
MSKE induces autophagy-mediated apoptosis. (A) C4-2 prostate cancer cells were treated with increasing concentrations of 0μ g/mL, 5 μg/mL, 10 μg/mL, and 20 μg/mL for 72 h. The cells were fixed with 4% paraformaldehyde and permeabilized with 0.1% sodium citrate and 0.1% Triton X. DNA fragmentation was determined by TdT-mediated dUTP nick end labeling (TUNEL). TUNEL assay (green channel). DAPI (blue channel) is used to locate the nuclei of the cells. (B) Cells were stained with Annexin V- Alexa Fluor 488 and PI and analyzed by flow cytometry following treatment with 5 μg/mL MSKE with or without 20 μM chloroquine. Percent of cells in the lower right quadrant that represent Annexin V ^+^/PI^-^ or early apoptotis and cells in the upper right quadrant that represent Annexin V ^+^/PI^+^ or late apoptotis was graphed. (C) Western blot analysis was performed to examine pro-apoptotic markers (Bax, cleaved caspase-3 and -7) and anti-apoptotic marker (BCL2) following treatment with MSKE in the presence or absence of 20 μM chloroquine. Actin was utilized as a loading control. (D) Results of western blot were analyzed using Image J and graphed. The experiments were performed in triplicate at least two times independently. Graphical data represents three independent experiments; * means 0.05 > *p* value > 0.01, ** means 0.01 > *p* value > 0.001, and *** means *p* value < 0.001.

To confirm that MSKE causes apoptosis, we treated the C4-2 cells with 5, 10, or 20 μg/ml MSKE and performed a TUNEL assay. Increased TUNEL staining (green) was observed with higher doses of MSKE (Fig 4A). These findings correlate with the Western blot analysis where up-regulation of pro-apoptotic markers PARP and caspase-12 was observed (Fig 2). These results support the hypothesis that MSKE induces apoptosis in C4-2 prostate cancer cells. To support the findings in the TUNEL assay and show that there was a correlation between autophagy and apoptosis, we performed quantitative apoptosis assay using flow cytometry *via* the Alexa Fluor 488 Annexin V in the presence or absence of chloroquine (autophagy inhibitor). Graphical representation of the apoptosis assay showed that treatment with MSKE led to an increase of apoptosis which was abrogated by treatment with chloroquine (Fig 4B). Western blot analysis confirmed these findings by showing that MSKE-mediated increase in pro-apoptotic proteins BAX, cleaved caspase-3 and -7, and decrease in anti-apoptotic protein BCL2 was abrogated by co-treatment with chloroquine (Fig 4C and 4D). Therefore, MSKE-mediated autophagy leads to apoptosis.

## Discussion

We investigated the impact of MSKE on the C4-2 proteome using a quantitative TMT isobaric labeling approach with subsequent protein identification by mass spectrometry. Changes in C4-2 cells were also examined by Western blot. Autophagy and apoptosis were determined by acridine orange, TUNEL/Annexin V analyses, respectively. The results indicated that MSKE significantly regulated components of the UPR stress inducing pathways that include chaperones, ER stress antioxidant enzymes, proteolytic enzymes, cytoskeletal proteins, as well as enzymes involved in autophagy or apoptosis. Autophagy is a dynamic process, whereby cytoplasmic proteins and cellular organelles are enveloped in autophagosomes and degraded by fusion with lysosomes for amino acid and energy recycling [17]. There is evidence that autophagy can play a critical role in cellular survival [18] but, it is frequently activated in tumor cells following anticancer therapies such as drug treatment and gamma-irradiation [19]. Apoptosis is defined as an active, fixed-pathway process of cell death characterized by cell shrinking, cytoplasmic condensation, ladder DNA degradation, and nuclear fragmentation resulting in the formation of apoptotic bodies. Initial studies on the mechanism of action of MSKE, suggest the ability to induce apoptosis via activation of the caspase cascade. The increase of apoptosis after MSKE treatment was confirmed with the TUNEL and Annexin V assays, in agreement with previous studies that have shown that MSKE promotes apoptosis in prostate cancer cells [7].

Our current study identified several proteins associated with apoptosis triggered by MSKE treatment such as STEAP2, PDCD6, MTDH, RAB5B, and cytochrome C oxidase (Table 1). STEAP2, also known as 6-transmembrane protein of the prostate 1 (STAMP1) is overexpressed in several types of human cancers, namely prostate, bladder, colon, pancreas, ovary, testis, breast, and cervix, but its clinical significance and role in cancer cells is still unclear [20]. STEAP2 participates in a wide range of biological processes, including molecular trafficking in the endocytic and exocytic pathways. It also controls cell proliferation and apoptosis. STEAP2 was down-regulated in C4-2 cells. It has been shown that STEAP2 blockage has a pro-apoptotic role that causes mitochondrial damage, and decreases cell proliferation and glucose uptake [20]. MTDH was up-regulated by 1.91 fold upon MSKE treatment in C4-2 cells. MTDH has been proposed to promote tumor progression through the integration of multiple signaling pathways including ras, myc, Wnt, PI3K/AKT, and NF-κB in various types of cancer [21–24].

The nuclear protein Apoptotic Chromatin Condensation Inducer 1 (ACIN1) is known to function in DNA fragmentation and is activated during apoptosis by caspase-3. It also plays an active role in spliceosome assembly because it is a critical subunit of an apoptosis and splicing-associated protein (ASAP) complex [25,26]. ACIN1 was down-regulated in our study by -1.60 fold and we speculate that MSKE induced an anti-apoptotic/survival response in C4-2 cells by down-regulating ACIN1. Taxilin protein (TXLNA), a binding partner of the syntaxin family that functions as a central player in the intracellular vesicle traffic, and is a marker of testicular injury [27] was also upregulated upon MSKE treatment.

Several results support the induction of apoptosis by MSKE via autophagy. The acridine orange staining showed that induced autophagy increased with MSKE treatment in a dose-dependent manner. Additionally, ER stress was significantly induced upon MSKE treatment in C4-2 cells as seen in the up-regulation of several stress-related proteins (e.g., GRP78, PDIA4, PDIA6, eIF2B, eIF4EBP1). We also identified COL4A3BP whose down-regulation sensitizes cancer cells to multiple cytotoxic agents, potentiating ER stress [28]. Lysosomal inhibitors such as chloroquine that inhibit acidification inside the lysosome or inhibit autophagosome-lysosome fusion and can block the degradation of LC3BII, leading to the accumulation of LC3BII, are good indicators for autophagy [29]. Co-treatment with MSKE plus chloroquine in C4-2 cells increased LC3B punctate staining as shown by immunofluorescence, and increased LC3-BII expression as shown by western blot analysis, proving that MSKE promotes autophagy which is blocked by chloroquine leading to accumulation of LC3BII. We speculated, however, that at 72 h we failed to see increased LC3BII with MSKE treatment alone due to high autophagic flux. This problem that sometimes arises when LC3BII expression does not increase when assayed at a certain time point as expected due to high autophagic flux is discussed in great detail with the conclusion that it is better to gauge autophagy by examining autophagosome accumulation in the presence of chloroquine [16]. We also demonstrated that chloroquine was able to reverse the extensive acridine orange leakage into the cytosol.

Degradation of cytosolic proteins in lysosomes is a hallmark of autophagy [30]. The absence of chromatin condensation is another characteristic of autophagy and indicative of apoptosis [31]. Recent studies have shown that a variety of anticancer therapies (including those that stimulate ER stress) activate autophagy in tumor cells, which has been proposed to either enhance cancer cell death or act as a mechanism of resistance to chemotherapy [32]. Autophagy works as a tumor suppression mechanism by removing damaged organelles/proteins, limiting cell growth and minimizing genomic instability [33]. Phosphatidylinositol 4-Kinase (PI4KA), a protein involved in both nonselective and selective types of autophagy [34], was up-regulated in MSKE-treated C4-2 cells. The expression of metadherin (MTDH), also known as astrocyte-elevated gene-1, a protein that may protect normal cells from serum starvation-induced death through protective autophagy [35], increased 1.91 fold in the presence of MSKE. In addition, phosphoglycerate kinase (PGK2), a cytosolic and glycolytic marker, was down-regulated by MSKE in C4-2 cells, suggesting that it may have been taken up into autophagic bodies [36].

ER stress was evident by the up-regulation of key ER stress markers like GRP78 and PDIA4, A6. Proper protein folding, maturation, and stabilization of the nascent protein in the ER requires a highly oxidizing ER environment, which is essential for the diverse post-translational and co-translational modifications (e.g., glycosylation, disulfide bridge formation) to which proteins are subjected after entering the ER. These processes are assisted and monitored by several resident chaperones and binding proteins, including glucose-regulated proteins like GRP78, which was up-regulated in the presence of MSKE. Folding enzymes, such as the thioredoxin-like protein (PDI), oxidize cysteine residues in nascent proteins (i.e., oxidative folding) resulting in the formation of intra- and intermolecular disulfide bonds. MSKE resulted in ER stress pathway activation and subsequent translation initiation component activation (i.e., eIF4EPB, eIF2). Moreover, the upregulation of pro-apoptotic markers MTDH, caspase 12, Cytochrome C oxidase, PDCD6 and NQO1, along with the increased apoptosis visualized by TUNEL and Annexin V staining, suggest that the ER stress response might eventually trigger apoptosis.

Overexpression of MTDH, also known as LYRIC, is observed in a variety of cancers and is involved in cancer initiation, proliferation, invasion, metastasis and chemoresistance [37]. MTDH also activates the PI3K/Akt pro-apoptotic pathway in cancer [38] and its inhibition induces apoptosis in prostate cancer cells [39]. Hudson et al. have also shown that treatment with MSKE activates the PI3K/Akt pro-apoptotic pathway in prostate cancer cells [7].

The upregulation of cytoplasmic C oxidase and Glyoxalase I (GLO1) suggests that MSKE also interferes with glycolysis and mitochondrial metabolism in C4-2 cells. GLO1 is a ubiquitous cellular defense enzyme involved in detoxification. GLO1 expression may protect cells against methylglyoxal-dependent protein adduction and cellular damage associated with diabetes, cancer, and chronological aging. GLO1 upregulation has been shown to play a pivotal role in glycolytic adaptations of cancer cells [40]. Phosphoglycerate kinase, a protein encoded by the *PGK1* and *PGK2* genes, is a glycolytic protein activated by MSKE. Phosphoglycerate kinase converts 1,3-diphosphoglycerate into 3-phosphoglycerate in the glycolysis pathway. *PGK1* is located on the X chromosome and is ubiquitously expressed whereas *PGK2*, whose differential expression was induced by MSKE in C4-2 cells, is a retrotransposed copy of *PGK1* located on chromosome 6 that shows a testis specific expression pattern [41,42]. Our analyses demonstrated overexpression of HYOU1, CTTN and DPYSL5, and down-regulation of ABCA5 proteins in C4-2 cells. These proteins have been implicated in other cancer cells and tumor types and are involved in relevant cell mechanisms for apoptosis evasion, increased tumor invasiveness, tumor hypoxia, cellular respiration, and mitochondrial fragmentation. ABCA5 is a member of the ATP-binding cassette transporter 1 subfamily of genes whose mutations are linked to several human genetic disorders including cystic fibrosis, neurological disease, retinal degeneration, cholesterol and bile transport defects, anemia, and drug response phenotypes [43].

MSKE also activates oxidative stress and ROS pathways through aldehyde dehydrogenases (ALD), sideroflexin, sulfiredoxin and DHRS1. ALDH belongs to a group of NAD(P)^+^-dependent enzymes involved in oxidation of a large number of aldehydes into their weak carboxylic acids [44]. ALDH is important for drug resistance, cell proliferation, differentiation, and response to oxidative stress in prostate cancer and its activity is used to distinguish between normal cells and their malignant counterparts. In a previous study, high ALDH activity was used to isolate human prostate cancer cells with significantly enhanced clonogenic and migratory properties both *in vitro* and *in vivo*. As seen in other cancer tissues, the percentage of ALDH cells in prostate cancer cell lines are also related to tumorigenicity and metastatic behavior [45].

MSKE induced apoptosis via the up-regulation of ER stress-driven caspase-3,-7 and -12. Treatment with chloroquine blocked the effects of MSKE on apoptosis by up-regulating BCL-2, decreasing BAX, preventing the cleavage of caspase-3 and -7 and antagonizing MSKE-mediated increase in early and late apoptosis. This further supports the findings that MSKE-mediated autophagy leads to apoptosis. MSKE also prompted the down-regulation of anti-apoptotic and survival proteins like Annexin A4 (ANXA4), a member of the Ca^2+^-regulated and phospholipid-binding annexin superfamily. ANXA4 expression is increased in many cancer types, including cancers of renal, gastric, colonic, ovarian, and cervical origins [46–50]. Its expression has been linked to loss of cell-to-cell adhesion, increased metastasis, and chemo-resistance, and it is considered a potential cancer diagnostic and therapeutic target [51,52]. *In vitro* studies suggest that ANXA4 exhibits an anti-apoptotic effect by activating NF-κB transcriptional activity [53,54]. Our data suggests that MSKE may induce apoptosis by decreasing ANXA4, and increasing MTDH.

**Fig 5:**
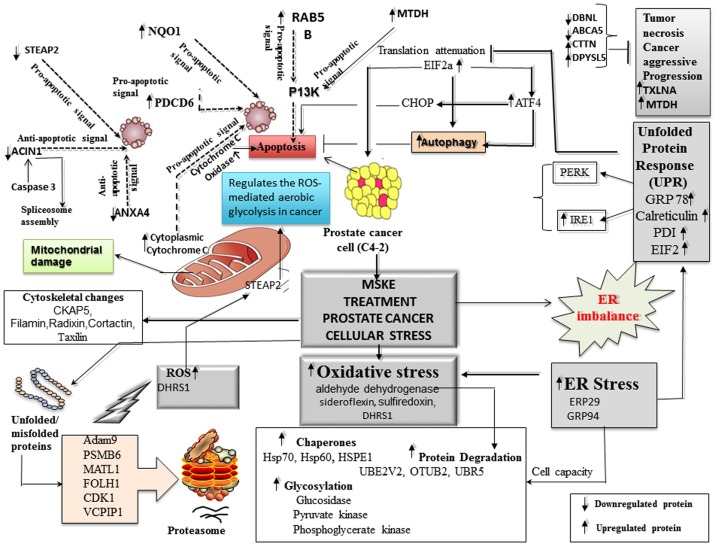
Proposed model highlighting unfolded protein response pathway with pro-apoptotic protein signatures triggered by ER stress in MSKE treated C4-2 prostate cancer cells. MSKE treatment of C4-2 cells promoted an unfolded protein response (UPR) pathway in a mitochondria-specific stress response (UPRmt) with pro- and anti-apoptotic protein signature triggered by ER stress. Strong ER stress and activation of the UPR initiate apoptosis. In contrast, mild UPR activation induces a beneficial ER homeotic response by reducing the load of misfolded proteins and by activating cellular protective mechanisms like autophagy. PERK mediates phosphorylation of eIF2α and ATF4-dependent transcriptional activation of autophagy proteins. The model highlights insufficient folding or degradation capacity in the mitochondria, contributing to apoptosis.

Recently, the cross-talk between autophagy and apoptosis has been considered as a key factor in the development and treatment of cancer [55]. The two pathways share molecular regulators and, in some cases, are activated by the same stimulus. Taken together, the results of this study suggest that MSKE induces apoptosis through signaling pathways that modulate ER stress, autophagy, cytoskeletal changes, cell-matrix, and cell-cell adhesion, as well as glycolysis and mitochondrial metabolism (Fig 5); opening the door to novel therapeutic and clinical exploitations.

## Supporting Information

S1 Table Differentially expressed proteins in MSKE-treated and control cultures. Detailed information including protein ID, gene name, number of amino acids, molecular weight, calculated pI, description, coverage samples, protein expression, change folds, and p-values.
(XLSX)
